# Transcriptomic and physiological analysis of the response of *Spirodela polyrrhiza* to sodium nitroprusside

**DOI:** 10.1186/s12870-024-04766-6

**Published:** 2024-02-08

**Authors:** Yamei Zhang, Rong Jia, Tanyue Hui, Yue Hu, Wenjing Wang, Yi Wang, Yong Wang, Yerong Zhu, Lin Yang, Beibei Xiang

**Affiliations:** 1 School of Chinese Materia Medica, Tianjin University of Traditional Chinese Medicine, Tianjin, 301617 P. R. China; 2 College of Life Science, Nankai University, Tianjin, 300071 China; 3 Tianjin Key Laboratory of Animal and Plant Resistance, College of Life Sciences, Tianjin Normal University, Tianjin, 300387 China

**Keywords:** *Spirodela polyrrhiza*, Sodium nitroprusside, Metabolic flux, Synthetic biology

## Abstract

**Background:**

*Spirodela polyrrhiza* is a simple floating aquatic plant with great potential in synthetic biology. Sodium nitroprusside (SNP) stimulates plant development and increases the biomass and flavonoid content in some plants. However, the molecular mechanism of SNP action is still unclear.

**Results:**

To determine the effect of SNP on growth and metabolic flux in *S. polyrrhiza*, the plants were treated with different concentrations of SNP. Our results showed an inhibition of growth, an increase in starch, soluble protein, and flavonoid contents, and enhanced antioxidant enzyme activity in plants after 0.025 mM SNP treatment. Differentially expressed transcripts were analysed in *S. polyrrhiza* after 0.025 mM SNP treatment. A total of 2776 differentially expressed genes (1425 upregulated and 1351 downregulated) were identified. The expression of some genes related to flavonoid biosynthesis and NO biosynthesis was upregulated, while the expression of some photosynthesis-related genes was downregulated. Moreover, SNP stress also significantly influenced the expression of transcription factors (TFs), such as ERF, BHLH, NAC, and WRKY TFs.

**Conclusions:**

Taken together, these findings provide novel insights into the mechanisms of underlying the SNP stress response in *S. polyrrhiza* and show that the metabolic flux of fixed CO_2_ is redirected into the starch synthesis and flavonoid biosynthesis pathways after SNP treatment.

**Supplementary Information:**

The online version contains supplementary material available at 10.1186/s12870-024-04766-6.

## Background

Duckweed plants are the smallest and simplest flowering aquatic plants in the world, being only a few millimetres in size. These tiny plants belong to the Lemnaceae family and Lemnoideae subfamily, which includes five genera: *Landoltia*, *Lemna*, *Spirodela*, *Wolffiella*, and *Wolffia* [1]. The plant body consists of a leaf-like structure (the frond), with or without a root-like structure. The plants propagate mainly via asexual reproduction and have a long yearly production period with an almost exponential growth rate, producing biomass faster than most other plants [2] and with a high content of starch [3] and protein [4] and a low content of lignocellulose [5]. In addition, they have extremely wide adaptability to different environments [6].

In recent years, *S. polyrrhiza*, as a chassis plant, has provided breakthroughs in biosynthesis. The advantages are as follows: (1) In terms of biological structure, *S. polyrrhiza*, the largest duckweed in North America, is approximately 7 to 15 mm long and has enormous fronds, making it useful for laboratory molecular biology research and tissue culture operations [7]. (2) This plant has a clear genetic background, the duckweed family's first species with a fully sequences genome was *S. polyrrhiza*, and a high-confidence genetic map of this plant has been created based on the genomic data [8]. (3) This plant is a well established genetic modification platform: to date, the genetic transformation of *S. polyrrhiza* has been achieved successfully, and the transformation efficiency has reached 5%-13% [9–11]. (4) This plant contains abundant precursor substances, including a variety of beneficial secondary metabolites, such as terpenoids and phenolic compounds [12].

Sodium nitroprusside (SNP), an established NO donor used in plant science research, simultaneously releases NO, cyanide (CN^−^) and Fe (II) in solution [13–15]. Research has demonstrated that SNP treatment improves the activity of antioxidants in blueberry fruit, tomato, soybean sprouts, okra, chard and alfalfa [16–21]. In recent years, research has shown that SNP participates in the biosynthesis of secondary metabolites such as alkaloids [22], flavonoids [23], terpenoids [24] and saponins [25]. The majority of these studies, however, concentrated on vegetables and herbaceous plants [26–28], and few studies have been conducted on aquatic plants [29].

Previous studies have shown that *S. polyrrhiza* copes with abiotic stress by fixing CO_2_ into the starch biosynthesis pathway; as a result, biomass rich in starch rapidly accumulates [30]. We are curious about how the energy distribution of *S. polyrrhiza* changes under these conditions; it remains unknown whether the metabolic flux is primarily directed into the starch or flavonoid biosynthesis branch after SNP treatment. In our work, *S. polyrrhiza* was treated with SNP, and the changes in biomass accumulation, starch content, flavonoid content, and antioxidant-related enzyme activity dynamics of *S. polyrrhiza* were studied. Moreover, we conducted research on the response of *S. polyrrhiza* to SNP stress using transcriptomic approaches. This provided molecular support for research on the metabolic flux in *S. polyrrhiza* under SNP treatment and the use of *S. polyrrhiza* as a bioreactor. This is crucial for the development of *S. polyrrhiza* as a model plant for synthetic biology.

## Result

### Photosynthetic pigment content and chlorophyll fluorescence

**Fig. 1:**
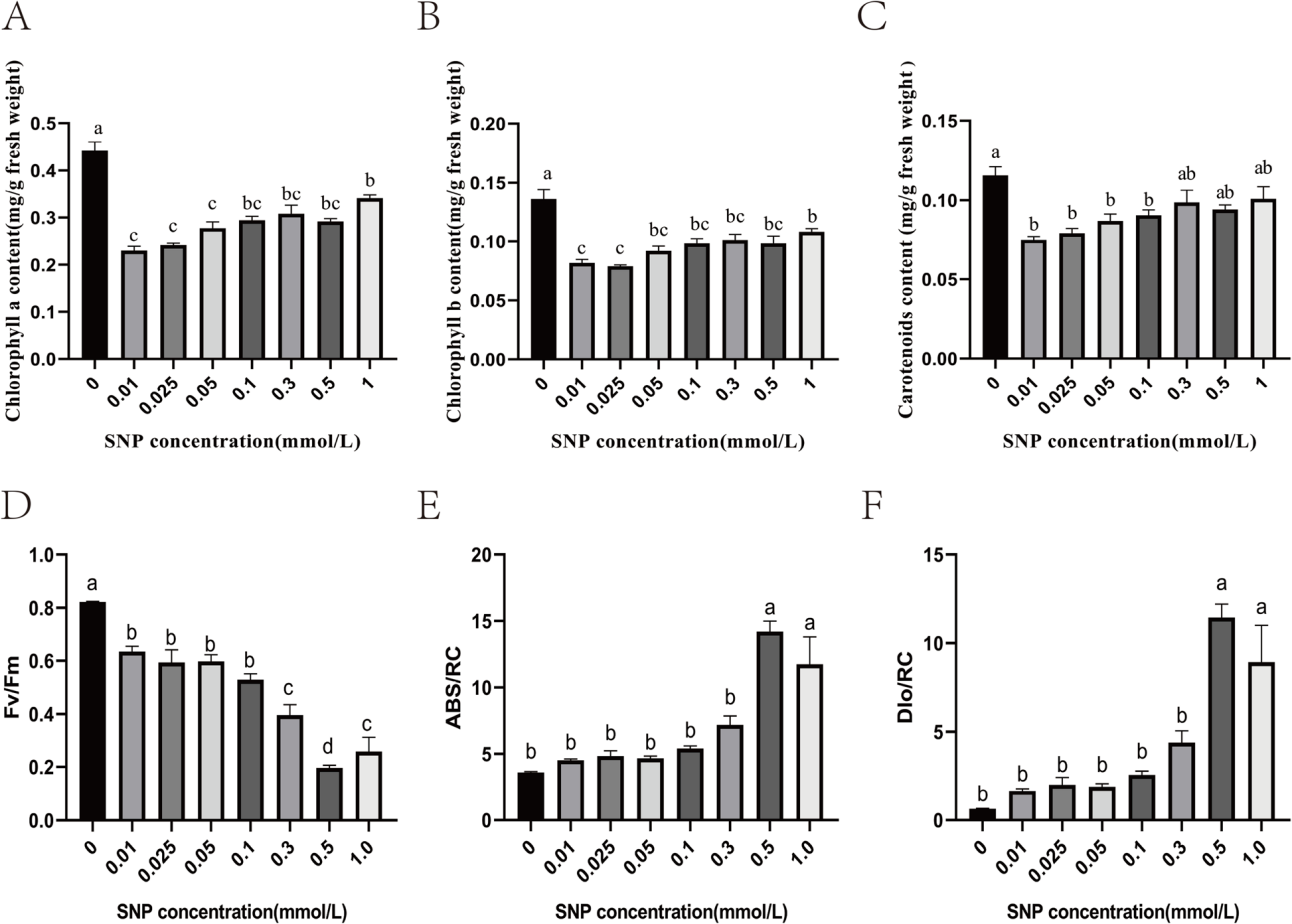
The effect of SNP stress on the photosynthetic pigment content and chlorophyll fluorescence of *S. polyrrhiza*. **A** chlorophyll a content; **B** chlorophyll b content; **C** carotenoid content; **D** Fv/Fm; **E** ABS/RC; **F** DIo/RC. The values are the means ± standard errors of triplicate assays. Bars with different lowercase letters are significantly different by one-way ANOVA (*P* < 0.05)

Quantification of chlorophyll is frequently used to estimate how plants react to various stresses. As the SNP concentration increased, the photosynthetic pigment content showed a rising trend after first decreasing. The chlorophyll a, chlorophyll b, and carotenoid contents were reduced by 48%, 39.7%, and 35.3% at 0.01 mM SNP in comparison to the control group, respectively (Fig. 1 A, B, C). As the SNP concentration increased (Fig. 1 D, E, F), the PSII maximum photochemical conversion efficiency (Fv/Fm) showed a downwards trend, being lowest at 0.5 mM SNP and decreasing by 76.2% compared with the control group. The absorption flux per reaction centre (ABS/RC) and dissipation energy per reaction centre (DIo/RC) change trends were contrary to that of Fv/Fm, peaking at 0.5 mM SNP and increasing by 2.94- and 16.9-fold compared with the control group, respectively.

### Effects of SNP treatment on the physiological changes of *S. polyrrhiza*

**Fig. 2:**
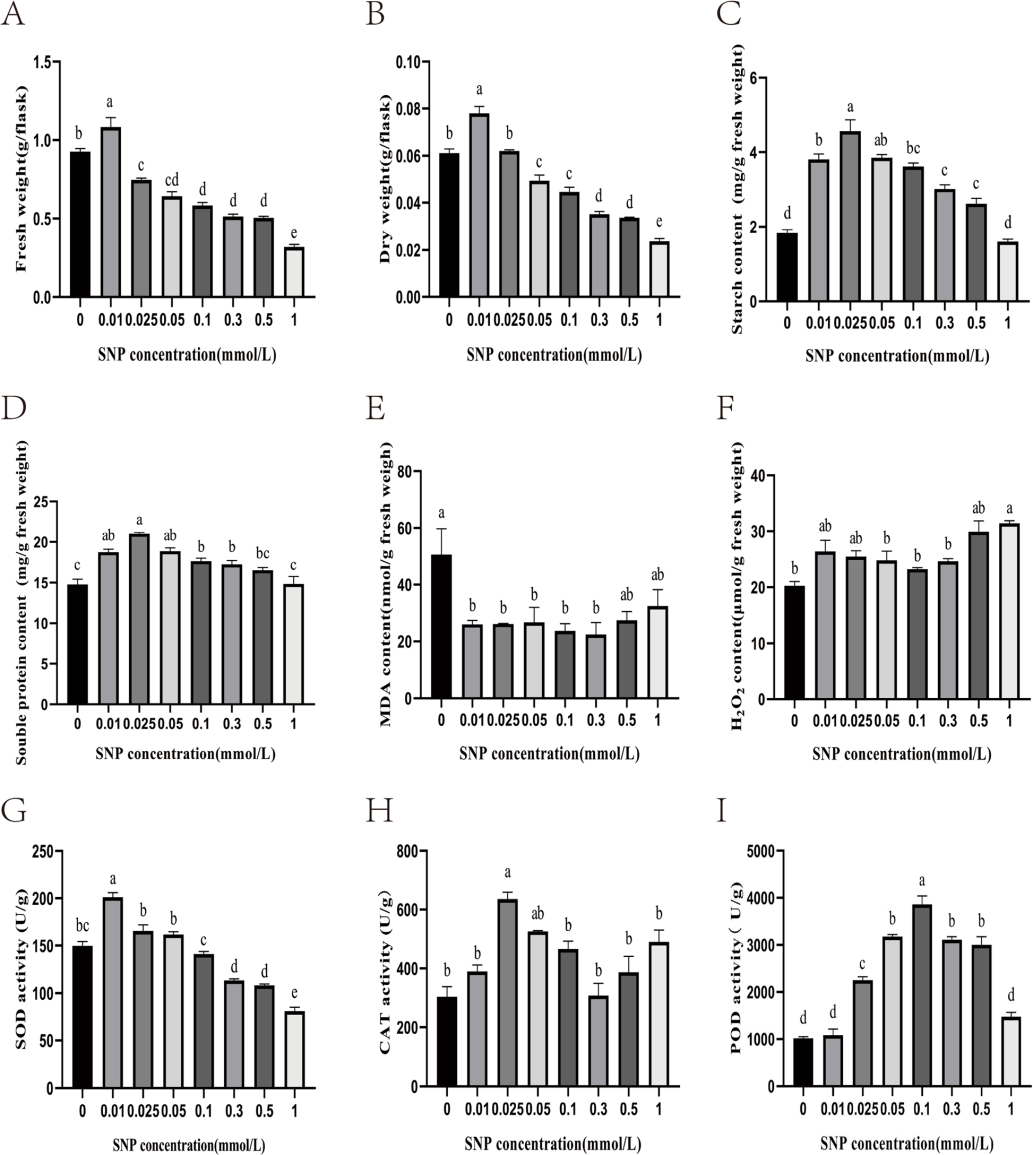
Effects of different concentrations of SNP on changes in the physiological and phytochemical characteristics of *S. polyrrhiza.* **A** fresh weight; **B** dry weight; **C** starch content; **D** soluble protein content; **E** MDA content; **F** H_2_O_2_ content; **G** SOD activity; **H** CAT activity; **I** POD activity. The values are the means ± standard errors of triplicate assays. Bars with different lowercase letters are significantly different by one-way ANOVA (*P* < 0.05)

As the SNP concentration increased, the fresh weight showed a significant downwards trend after rising first and peaked at 0.01 mM SNP, increasing by 12.58% compared with the control group (Fig. 2A). Similar to the fresh weight trend, the dry weight of *S. polyrrhiza* showed an increase of 27.87% in the 0.01 mM SNP treatment samples compared with the control group (Fig. 2B). The starch content change trend was consistent with those of the fresh and dry weight; the starch content increased and then decreased, peaking at 0.025 mM SNP, which was approximately 1.5 times more than that in the control (Fig. 2C). The soluble protein change trend was consistent with that of starch. The highest soluble protein content was demonstrated in plants grown on medium with 0.025 mM SNP, increasing by 42.06% over the control group (Fig. 2D). The malondialdehyde (MDA) content was lower in the SNP treatment group than in the control group, with the lowest significant MDA content observed at 0.3 mM SNP, decreasing by 55.63% (Fig. 2E), which implied that exogenous NO at a specific concentration effectively inhibited the accumulation of MDA. Compared with the control group, the hydrogen peroxide (H_2_O_2_) content of the 1.0 mM SNP-treated group increased from 20.25 mol/g FW to 31.41 mol/g FW. Excessive accumulation of H_2_O_2_ may further aggravate membrane lipid peroxidation (Fig. 2F). The superoxide dismutase (SOD) activity showed a significant downwards trend after first increasing. Compared to the control group, the SOD activity of the 0.01 mM SNP treatment group increased rapidly, from 149.67 U/g FW to 201.67 U/g (Fig. 2G). The catalase (CAT) activity peaked at 0.025 mM SNP and was 108% higher than that in the control group (Fig. 2H). The peroxidase (POD) activity showed a significant downwards trend after rising first with increasing SNP concentration and was significantly higher in the 0.1 mM SNP treatment group (3860.03 U/g) than in the control group (1015.43 U/g) (F ig. 2I).

### Effects of SNP treatment on the flavonoid content of *S. polyrrhiza*

**Fig. 3:**
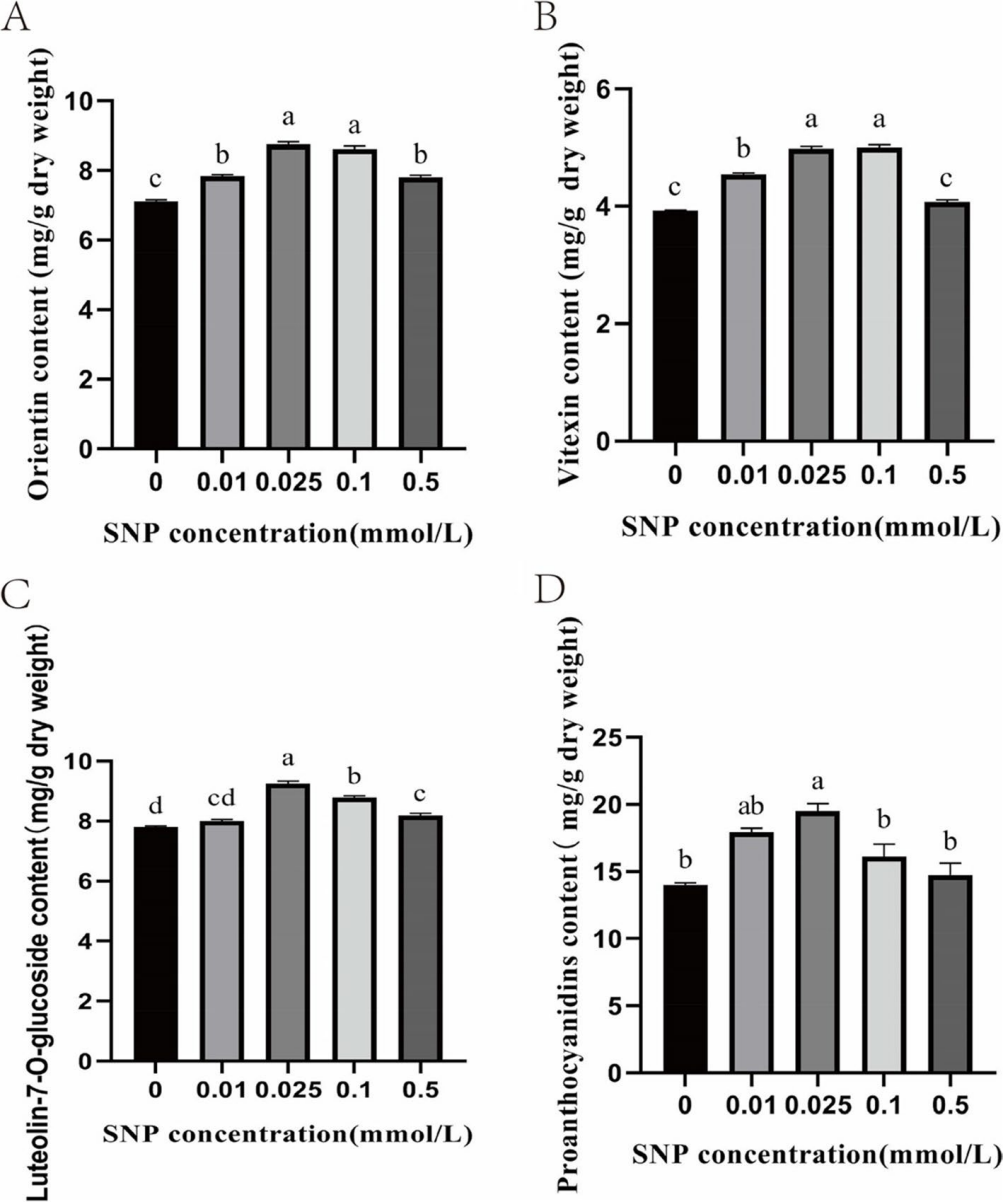
Effects of different concentrations of SNP on the flavonoid content of *S. polyrrhiza*. A orientin content; B vitexin content; C luteolin-7-O-glucoside content; D proanthocyanidin content. The values are the means ± standard errors of triplicate assays. Bars with different lowercase letters are significantly different by one-way ANOVA (*P* < 0.05)

Previous studies have reported that the duckweed species *S. polyrrhiza* has been widely utilized as folk medicine in China, Korea and a few European nations [31]. Duckweeds are medicinal herbs that do not have severe side effects [32]. Flavonoids, such as apigenin, orientin, vitexin, and luteolin-7-O-glucoside, are the main pharmacological components of duckweed [33]. Orientin, vitexin and luteolin-7-O-glucoside have anti-inflammatory, antioxidant, antitumour and other effects [34–36]. We determined the levels of three bioactive compounds of the flavonoid biosynthesis pathway: orientin, vitexin, and luteolin-7-O-glucoside. The orientin content showed a significant downwards trend after rising first, peaking at 0.025 mM SNP and increasing by 18.9% compared with the control group (Fig. 3A). The vitexin content significantly increased at SNP treatment concentrations ranging from 0.01 mM to 0.1 mM compared to the control group (Fig. 3B). The luteolin-7-O-glucoside content significantly increased at SNP treatment concentrations ranging from 0.025 mM to 0.5 mM and peaked at 0.025 mM SNP, and the value was 15.8% higher than that in the control group (Fig. 3C).

Proanthocyanidins are a kind of polyphenol. Their special structure determines their strong antioxidation activity, and they have anticancer, antimutagenic, cardiovascular protection and other effects [37]. The proanthocyanidin content showed a downwards trend after rising first with increasing SNP concentration. The maximum value was reached at 0.025 mM SNP (Fig. 3D). This result is consistent with the observation that at 0.01 and 0.025 mM SNP, leaves exhibited chlorosis and accumulation of purplish red pigment on the back (Additional file 1: Figure S1).

In this study, we wanted to explore the redirection of metabolic flux from fixed CO_2_ into starch or flavonoid biosynthesis branches after SNP treatment. Therefore, we focused on the changes in starch and flavonoid contents and found that under 0.025 mM SNP treatment, the starch and flavonoid contents increased significantly. Based on these results, *S. polyrrhiza* treated with 0.025 mM SNP was selected for use in subsequent RNA expression analyses.

### Global gene analysis in *S. polyrrhiza* after 0.025 mM SNP treatment

In total, six cDNA libraries were constructed and sequenced. While robust data were collected, after data filtering, 43.50, 44.84, 44.32, 39.85, 48.41, and 39.08 million high-quality reads were obtained in the CK and SNP treatments, respectively. Subsequently, these clean reads were de novo assembled into 33,684 genes, with a mean length of 1,360 bp and an N50 length of 2,791 bp (Table 1).

**Table 1:** Characteristics of the de novo assembled transcriptome in *S. polyrrhiza* after 0.025 mM SNP treatment

Genes Num	GC percentage	N50 number	N50 length	Max length	Min length	Average length	Total assembled bases
33684	48.9927	5252	2791	18736	201	1360	45841369

**Fig. 4:**
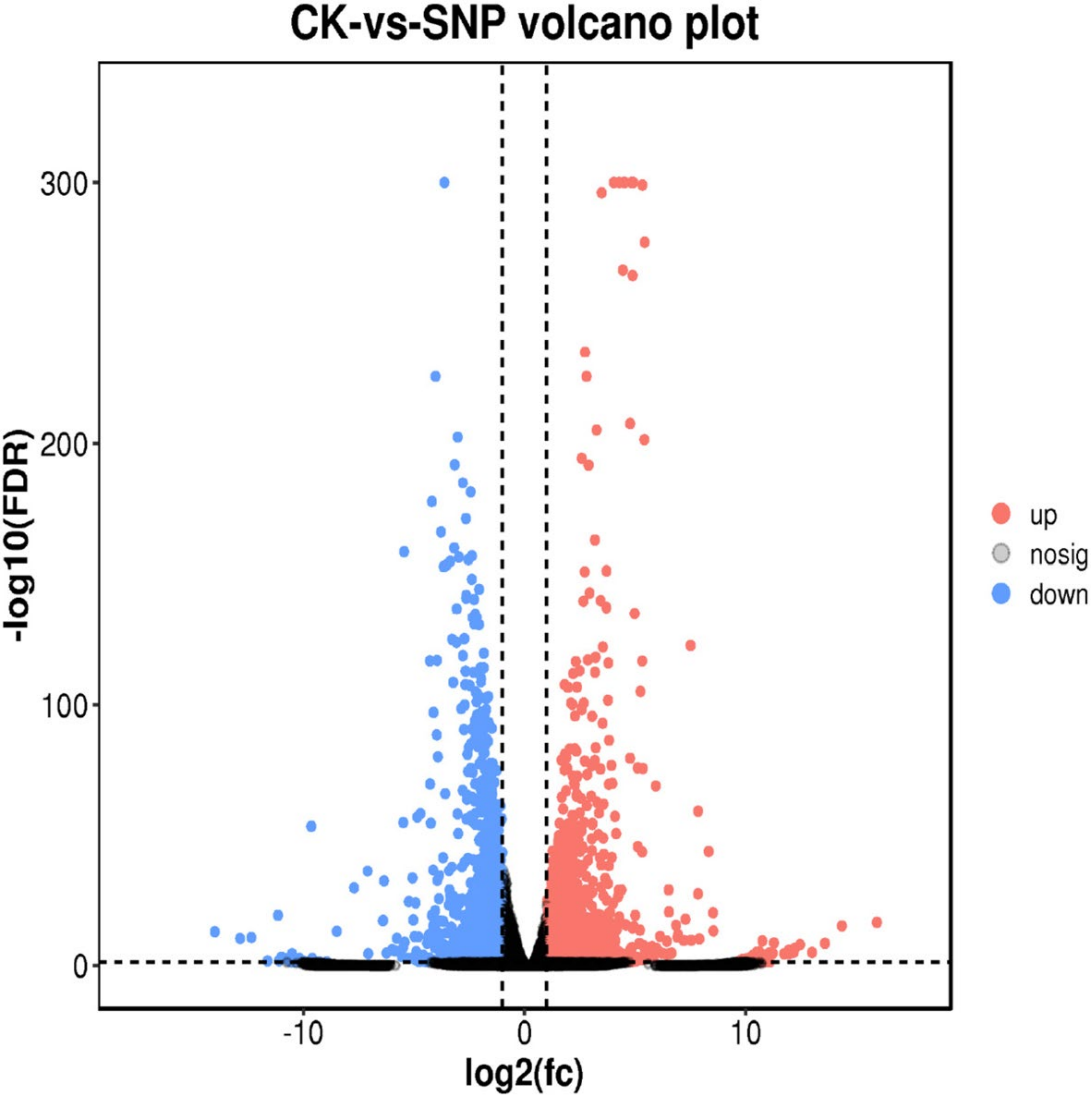
Volcano plots of the differentially expressed genes (DEGs) in *S. polyrrhiza* after 0.025 mM SNP treatment. The red dots indicate upregulated genes with significant differences, and the blue dots indicate downregulated genes with significant differences

The unigenes mapped to the database searches are shown in Table 2. Using the National Center for Biotechnology Information (NCBI) NR database, 15,127 unigenes were mapped to ten identified plant species and ‘other’ plant species (Additional file 2: Figure S2). Using the SwissProt database, 11,816 unigenes were annotated and reviewed with the UniProt Knowledgebase. Searches against the KOG database led to 9,341 unigenes being divided into 25 categories. Using the Kyoto Encyclopedia of Genes and Genomes (KEGG) database, 14,966 unigenes were enriched in 137 biochemical pathways, such as plant‒pathogen interaction, carbon metabolism, plant hormone signal transduction and phenylpropanoid biosynthesis. Transcriptome profiles were compared between the CK and SNP - treated groups. A total of 2,776 DE genes were identified, of which 1,425 were upregulated, while 1,351 were downregulated (Fig. 4).

**Table 2:** Functional annotation of unigenes in the NR, SwissProt, KEGG, and COG databases

BLASTx search against specific platforms	Values
NR	15127
SwissProt	11816
KOG	9341
KEGG	14966

### Investigation of differentially expressed transcription factors under SNP treatment

**Fig. 5:**
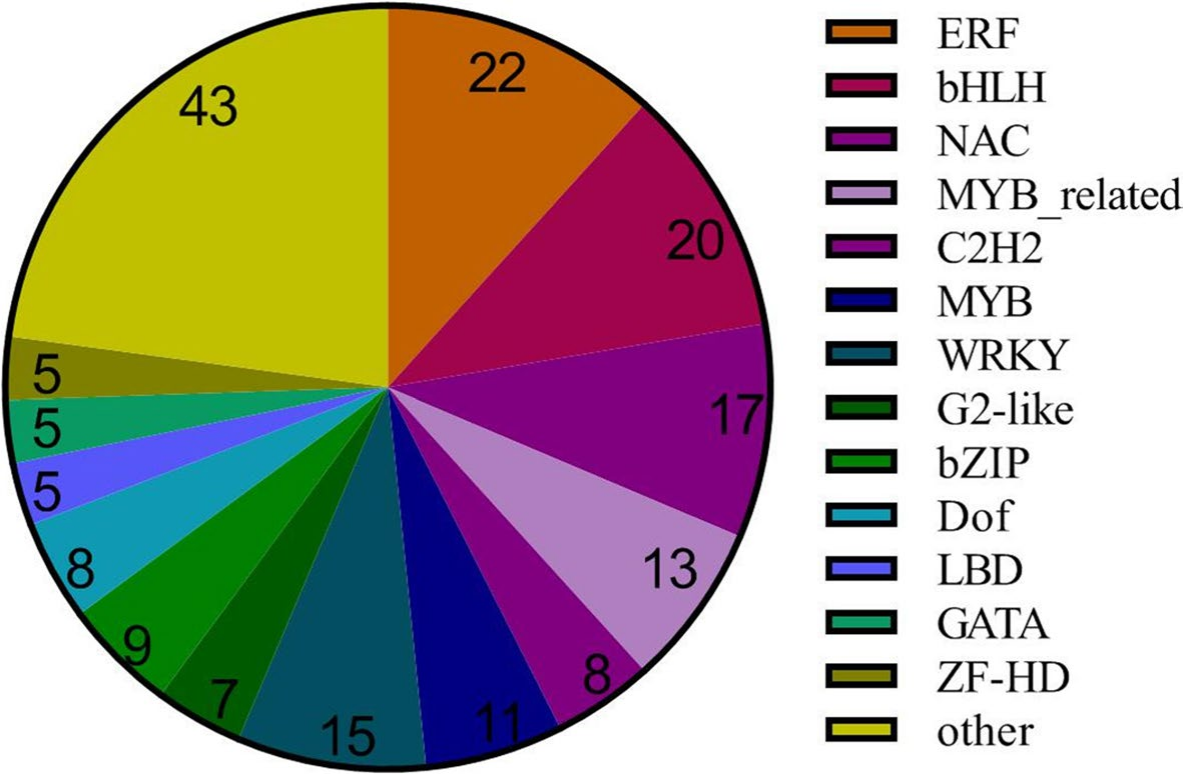
Classification of differentially expressed transcription factors in *S. polyrrhiza* after 0.025 mM SNP treatment

**Fig. 6:**
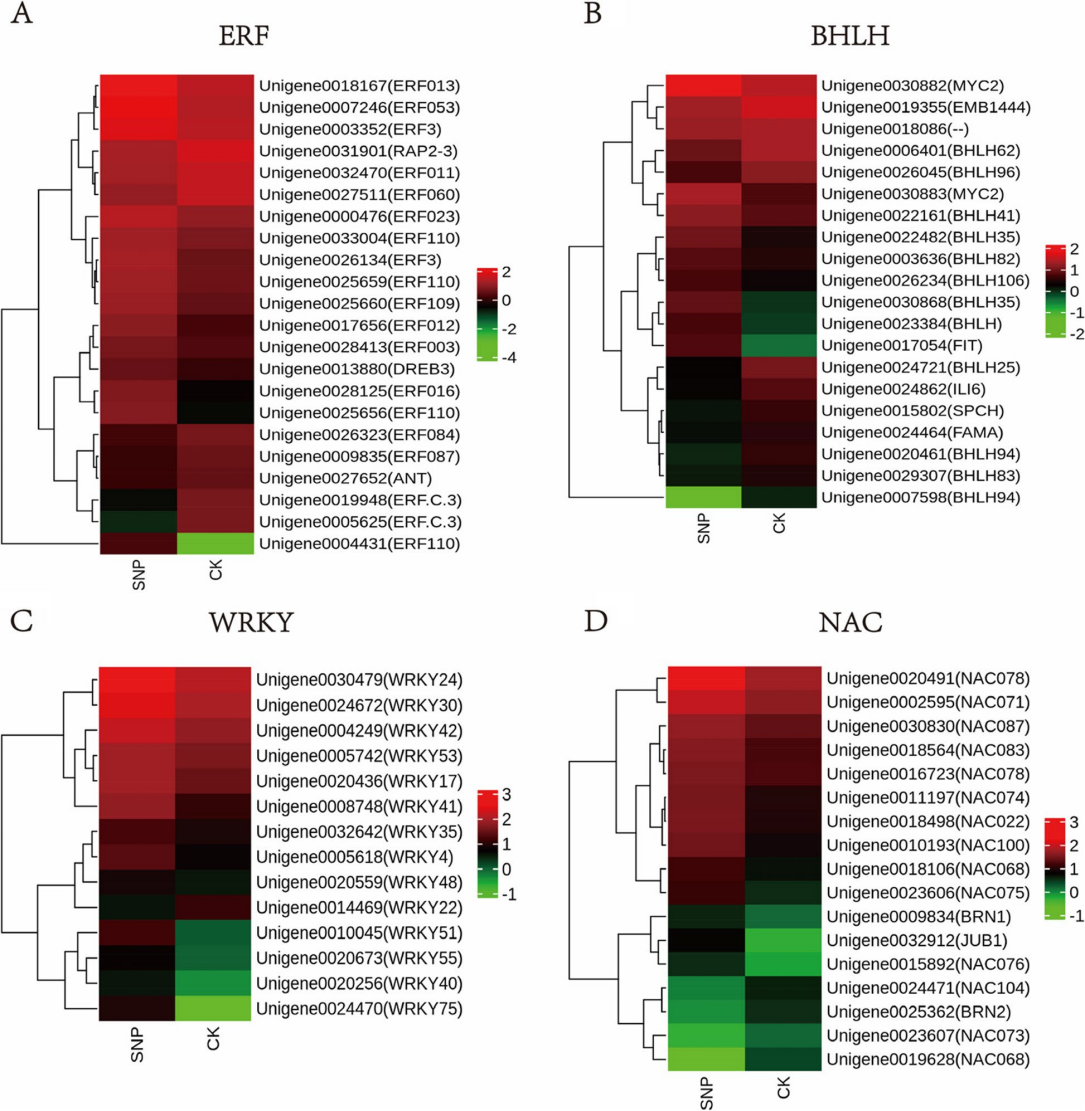
Expression pattern of genes associated with TFs under SNP treatment in *S. polyrrhiza*. **A** ERF family; **B** BHLH family; **C** WRKY family; **D** NAC family. The expression level was Z score normalized. The colour gradient (green to red) corresponds to the gene expression levels (low to high). Each row represents a gene, with its name given in parentheses

Transcription factors (TFs) are key regulators that temporarily and spatially turn on or off the transcription of their target genes by binding certain upstream elements [38]. The de novo assembly data identified 818 TFs. Among these factors, 208 TFs (125 upregulated and 83 downregulated) exhibited significant differences in expression levels in the 0.025 mM SNP treatments (Fig. 5). In this study, several TFs responded significantly to SNP stress (Fig. 6), including ERF (22), BHLH (20), NAC (17), and WRKY (15) (Additional file 4: Table S1). ERFs are specific to plants and play a role in a variety of developmental processes, such as flowering [39], seed development [40], and fruit ripening [41]. Among these TFs, the most abundant were those of the ERF family, which accounted for 11.7% of the total TFs. The RNA-Seq results showed that the expression of 15 ERF-related genes was upregulated and that of 8 genes was downregulated. BHLH TFs are involved in stress tolerance, pathogen defence, and nutrient uptake but also play an important role in the production of secondary metabolites such as flavonoids and anthocyanins [42, 43]. Twenty BHLH TFs were differentially expressed between the SNP concentrations; ten of these genes were upregulated, and ten were downregulated. A crucial part of plant biological and abiotic stress responses is played by NAC TFs, which are unique to plants [44]. Of the seventeen members of the NAC TF family, thirteen were upregulated, and four were downregulated. WRKY TFs participate in the response to injury, ageing, development, and disease [45]. Additionally, WRKY TFs control how plants grow and develop as well as how they react to stress. Fourteen of the fifteen members of the WRKY TF family were upregulated, while one gene was downregulated.

### Investigation of differentially expressed photosynthesis-related genes under SNP treatment

**Fig. 7:**
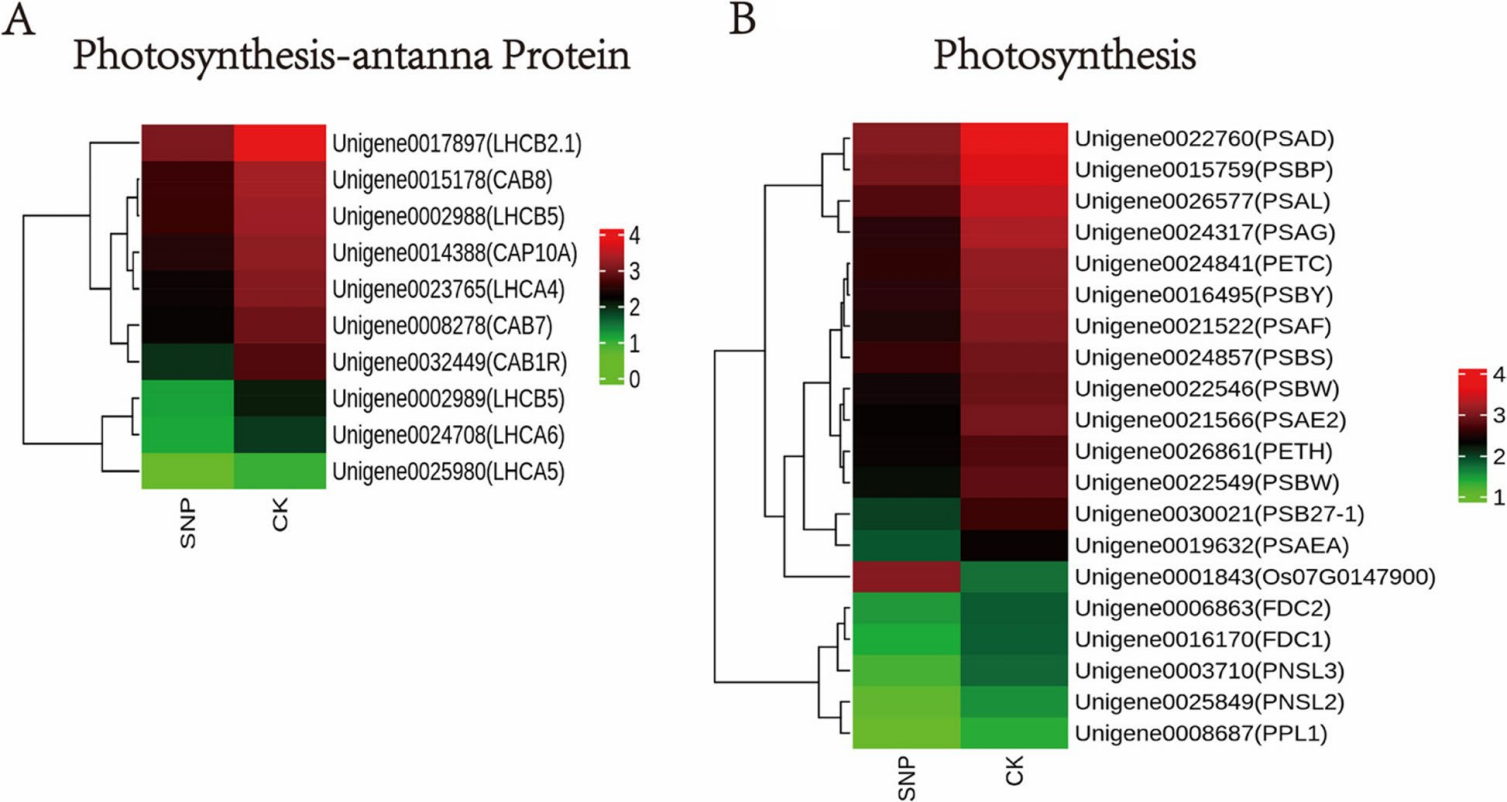
Heatmap displaying the expression levels of differentially expressed genes (DEGs) related to photosynthesis in *S. polyrrhiza*. **A** Expression pattern of genes involved in photosynthesis-antenna proteins under SNP treatment in *S. polyrrhiza*; **B** Expression pattern of genes involved in photosynthesis under SNP treatment in *S. polyrrhiza*. The expression level was Z score normalized. The colour gradient (green to red) corresponds to the gene expression levels (low to high). Each row represents a gene, with its name given in parentheses

Based on their functions, the genes involved in photosynthesis were selected for this study and classified into two groups (Fig. 7). Most of the photosynthesis-related genes belong to the first class, which is involved in the light reaction, such as the photosystem I and II reaction centre genes *PsbP*, *PsbQ*, *PsbS*, *PsbW*, *PsbY*, *Psb27*, *PsaD*, *PsaE*, *PsaF*, *PsaG*, *PsaL*, *PsaO* and *cytochrome b/6 complex PetC*, which were expressed at lower levels in the SNP treatment (Additional file 5: Table S2). The second category consists of antenna proteins, which are light-harvesting chlorophyll a/b binding (LHC) proteins. Most of the genes (including *Lchas* and *Lchbs*, which are light-harvesting protein genes) showed similar expression patterns to the light reaction genes (Additional file 5: Table S2).

### Investigation of differentially expressed starch metabolism-related genes under SNP treatment

**Fig. 8:**
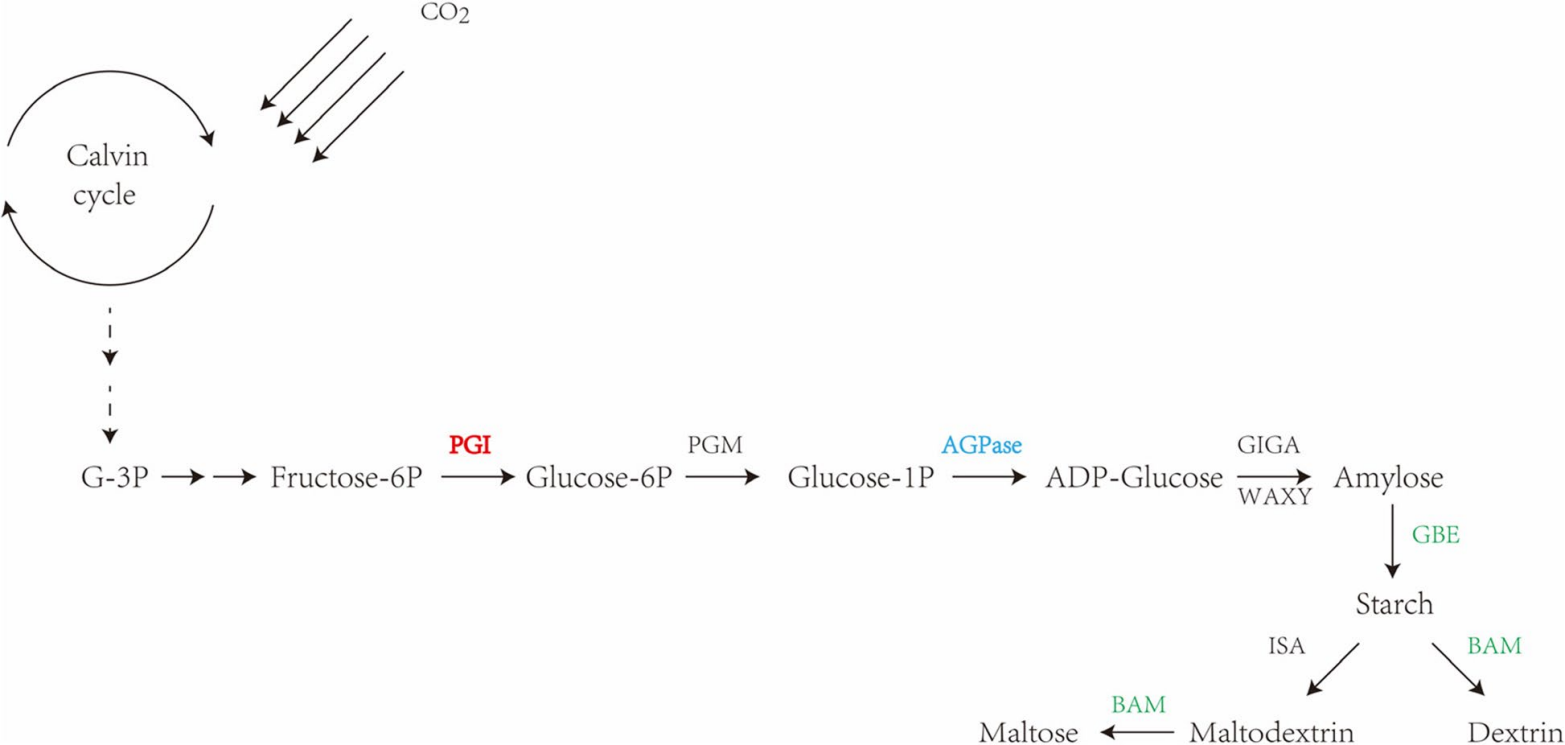
Expression pattern of genes involved in starch metabolism under SNP treatment in *S. polyrrhiza*. The arrows indicate the directions of catalytic reactions or transport. Red indicates upregulated expression, green indicates downregulated expression, and blue indicates up/downregulated expression. Dotted arrows indicate omitted steps. G-3P, glyceraldehyde 3-phosphate; fructose-6P, fructose 6-phosphate; glucose-6P, glucose 6-phosphate; PGI, glucose-6-phosphate isomerase; glucose-1P, glucose 1-phosphate; AGPase, ADP-glucose pyrophosphorylase; ADP-glucose, adenosine- 5-diphosphoglucose; SBE, starch branching enzyme; BAM, beta-amylase

Transcriptome data indicated that carbon fixation pathways were significantly affected by 0.025 mM SNP treatment. Following SNP treatment, the expression of a few genes related to the Calvin cycle was reduced. The SNP-downregulated genes included those encoding sedoheptulose-bisphosphatase (*SEBP*), fructose-bisphosphate aldolase (*ALDO*), fructose-1,6-bisphosphatase (*FBP*), ribose 5-phosphate isomerase A (*RPI*), ribulose-bisphosphatecarboxylase (*RBCS*), glyceraldehyde-3-phosphate dehydrogenase (GAPDH), and phosphoribulokinase (*RPK*). Starch metabolism is important for the supply of energy during *S. polyrrhiza* growth and development. Twenty-three DEGs were enriched in starch and sucrose metabolism pathways (Additional file 6: Table S3). Numerous important enzymes are involved in starch biosynthesis, such as ADP-glucose pyrophosphorylase (AGP). Half the transcripts encoding AGPase were upregulated, while the other half were downregulated (Fig. 8). The results showed that under SNP treatment, the expression of a transcript encoding AGPase (Unigene0007838) was upregulated from 35 to 90 FPKM, while the expression of another transcript encoding AGPase (Unigene0019277) was downregulated from 99 to 38 FPKM. This may be due to differences in gene expression regulation, which lays the foundation for the selection of target enzymes for further research in the future. The degradation of starch is catalysed by α-amylase and β-amylase [46], which are responsible for the degradation of starch to smaller hydrocarbons. β-amylase was downregulated under SNP treatment, while the expression of α-amylase did not change significantly. A transcript encoding β-amylase exhibited an expression level of 69.8 FPKM in the control group, and the value decreased to 28.5 FPKM under SNP treatment.

### Investigation of differentially expressed reactive oxygen species (ROS) homeostasis -related genes under SNP treatment

**Fig. 9:**
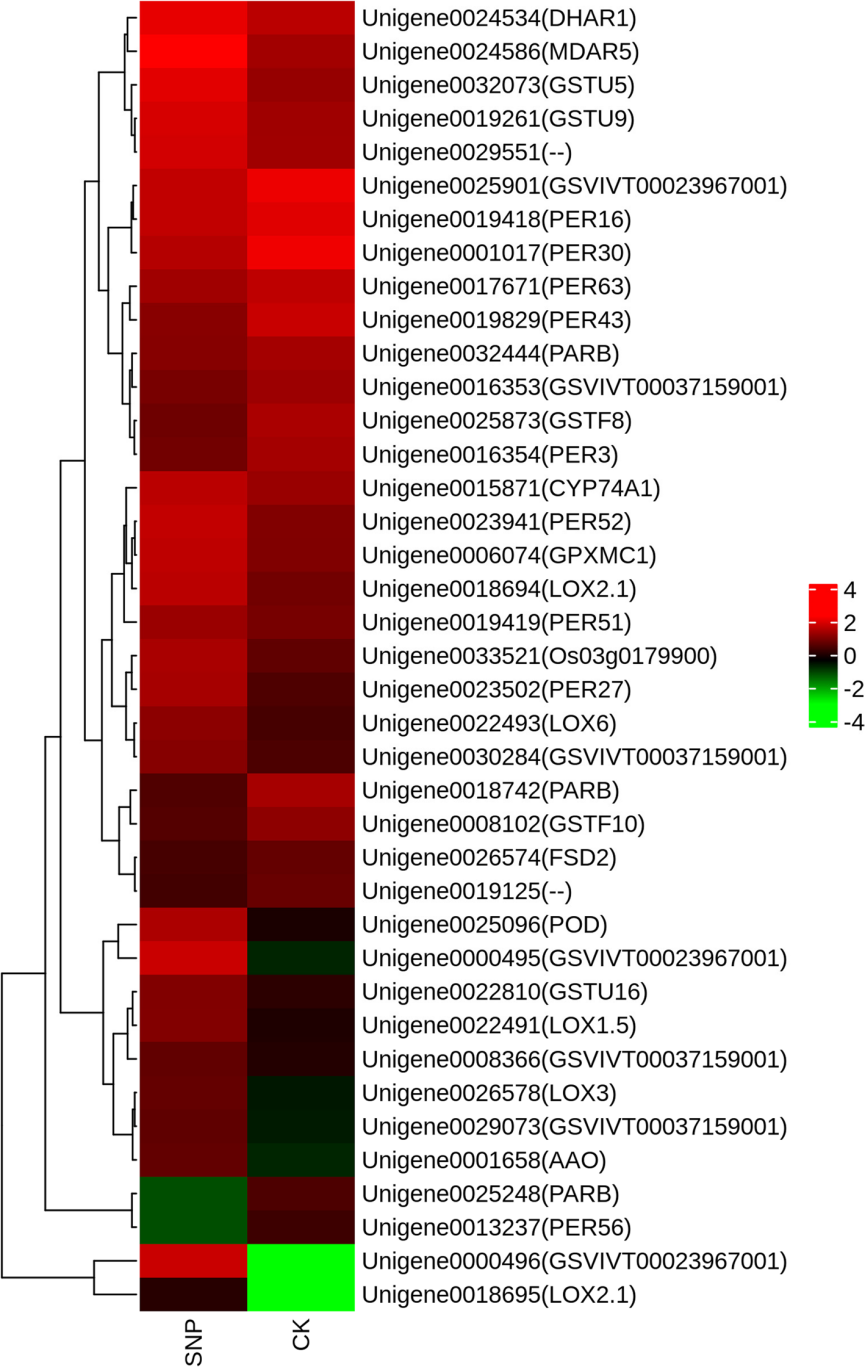
Expression pattern of genes involved in antioxidant activity under SNP treatment in *S. polyrrhiza*. The expression level was Z score normalized. The colour gradient (green to red) corresponds to the gene expression levels (low to high). Each row represents a gene, with its name given in parentheses

ROS are chemically reactive molecules that contain oxygen, and carbohydrates, lipids, proteins and DNA and are all damaged by the overproduction of ROS [47, 48]. The data from our transcriptome analysis revealed that the transcript abundance of genes encoding different components of the ROS scavenging machinery differed dramatically between the control and SNP treatments (Fig. 9). A total of 34 putative genes were found encoding proteins with antioxidant properties (Additional file 7: Table S4). There were 15 upregulated unigenes that encoded proteins such as glutathione s-transferases (*GST*), glutathione peroxidase (*GPX*), ascorbate peroxidase (*APX*), lipoxygenase (*LOX*) , monodehydroascorbate reductase (*MDAR*), and alternative oxidase (*AOX*). There were 6 downregulated unigenes that encoded proteins such as *SOD* and *GST*. Nine of the seventeen POD-encoding transcripts were upregulated, while eight were downregulated. However, there were no appreciable variations in the expression of CAT-encoding transcripts under SNP treatment compared to the untreated samples.

### Investigation of differentially expressed flavonoid biosynthesis-related genes under SNP treatment

**Fig. 10:**
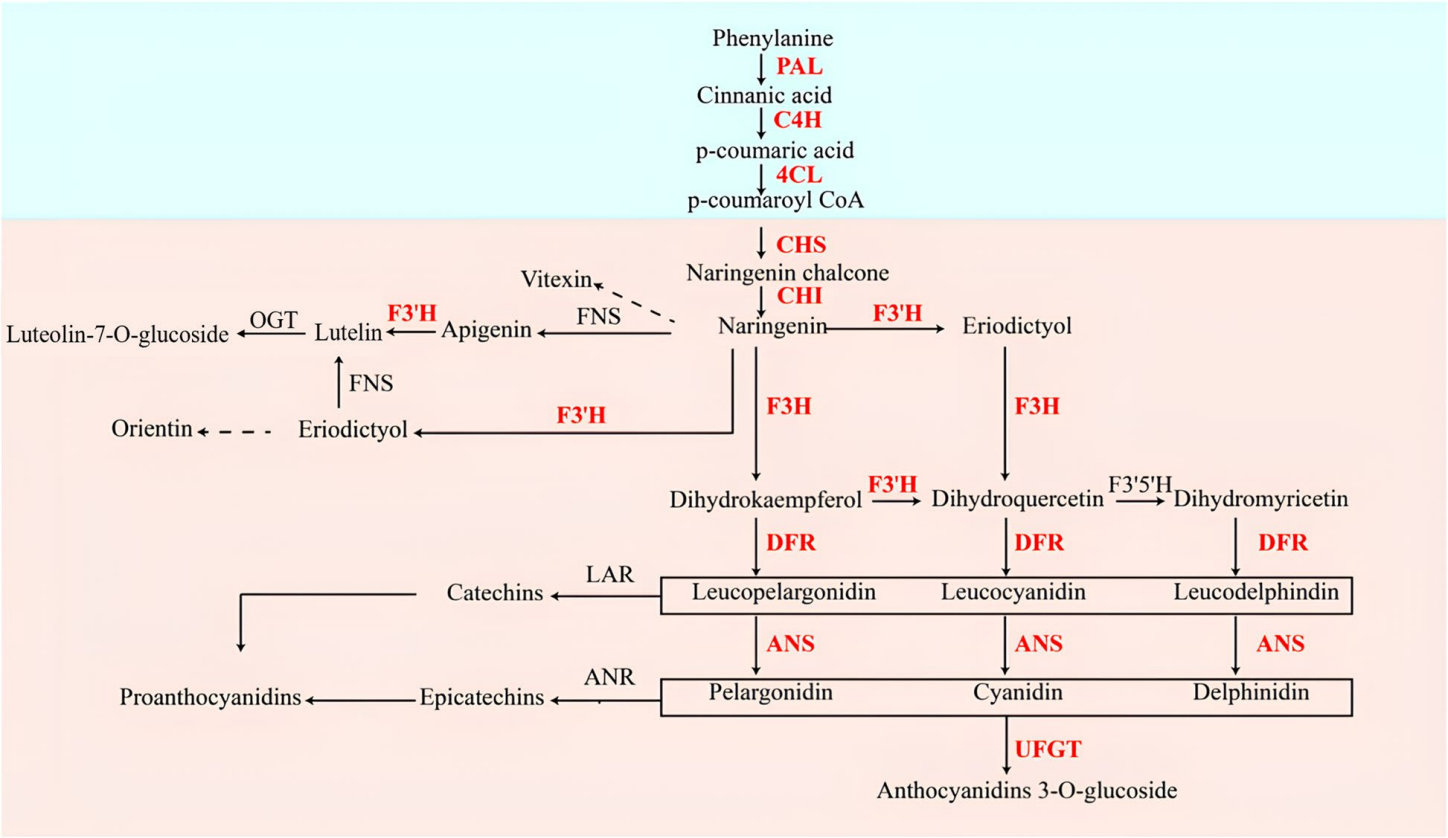
Biosynthetic pathway of flavonoids in *S. polyrrhiza*. Heatmap showing the changes in transcripts involved in flavonoid biosynthesis. Upregulated genes are shown in red. PAL, phenylalanine ammonium lyase; C4H, cinnamate 4-hydroxylase; 4CL, 4-coumarate-CoA ligase; CHS, chalcone synthase; CHI, chalcone isomerase; F3H, flavanone-3-hydroxylase; F3′H, flavonoid-3′-hydroxylase; DFR, dihydroflavonol reductase; ANS, anthocyanin synthase; UFGT, anthocyanidin 3-O-glucosyltransferase

The phenylpropanoid biosynthesis route in plants contributes to numerous different biosynthetic branches, such as flavonoid biosynthesis and anthocyanin biosynthesis (Fig. 10). Analysis of the *S. polyrrhiza* flavonoid synthesis pathway identified 13 differentially expressed structural genes (Additional file 8: Table S5). Phenylalanine ammonialyase (PAL), cinnamate 4-hydroxylase (C4H) and 4-hydroxycinnamoyl-CoA ligase (4CL) are universal factors involved in flavonoid biosynthesis [49]. The expression of transcripts encoding *PAL*, *C4H*, and *4CL* was upregulated 3.5-, 1.8-, and 2.8-fold, respectively, after SNP treatment. Chalcone synthase (CHS), chalcone isomerase (CHI) and flavanone 3-hydroxylase (F3H) are the enzymes that catalyse the first three reactions of the flavonoid biosynthesis branch. The expression of transcripts encoding *CHS*, *CHI* and *F3H* was upregulated 1.5-, 1.4- and 1.8-fold, respectively, after SNP treatment. Meanwhile, the expression of flavonoid-3′-hydroxylase (*F3′H*), dihydroflavonol 4-reductase (*DFR*), anthocyanidin synthase (*ANS*) and anthocyanidin 3-O-glucosyltransferase (*UFGT*) was upregulated 1.1-, 3.2-, 2.6- and 2.8-fold, respectively, after SNP treatment.

### Quantitative real-time PCR (qRT-PCR) validation

To further confirm the veracity and reliability of the DEG data generated by RNA-Seq, 17 DEGs (Additional file 9: Table S6) were selected for qRT‒PCR verification, including genes related to the flavonoid biosynthesis pathway, NO biosynthesis pathway, photosynthesis and TFs. The results showed that gene expression (17) was consistent with the RNA-Seq data (Additional file 3: Figure S3).

## Discussion

It has been reported that SNP stress probably triggers the NO biosynthesis pathway, resulting in a high endogenous NO level [19, 50]. To date, the main NO synthesis pathways identified are the NOS (nitric oxide synthase) and NR (nitrate reductase) pathways [51]. The NOS pathway is mainly dependent on NO synthase (NOS), which generates NO and L-citrulline by oxidizing L-arginine [52]. In the NR pathway, nitrate reductase (NR) can reduce NO_3_^−^ to NO_2_^−^, nitrite reductase (NiR) can reduce NO_2_^−^ to NO, and NR can also oxidize NO to nitrate to scavenge NO, which shows that NR is an important regulator of NO homeostasis in plants [51]. In the transcriptomic analysis of the key enzymes for NO synthesis, the *NRT* and *NR* genes exhibited increased expression under SNP stress (Additional file 10: Table S7). There is evidence that NO also increases the cytosolic level of free Ca^2+^ through upregulation of Ca^2+^ channels, transporter proteins, cGMP and cADPR [53, 54]. In the transcriptomic analysis of calmodulin expression, the genes encoding calmodulin, calcium-binding protein and the cyclic-nucleotide gated channel were upregulated, indicating that Ca^2+^ was regulated in *S. polyrrhiza* under SNP stress. Notably, every signalling pathway engages protein kinases, which can activate a variety of downstream TF family proteins [55]. In this study, several TFs responded significantly to SNP stress, including ERF (22), BHLH (20), NAC (17), and WRKY (15). However, more research is needed to determine whether these TFs work independently or in concert. A series of downstream NO-responsive genes are expressed as a result of TF activation, and their transcription further controls the equilibrium of cellular metabolism.

The most significant photosynthetic pigment in plant chloroplasts is chlorophyll (Chl), which is crucial for absorbing light and transmitting light energy in antenna systems [56]. The findings of this study showed that SNP treatment caused the chlorophyll content of *S. polyrrhiza* to decrease, and similar findings were seen in other investigations, such as in *Torreya grandis* [57], chard [20] and soybean seedlings [58]. This may be due to the damage to photosynthetic pigments caused by SNP stress or the degradation of chlorophyll induced by NO [59]. In addition, there are also reports indicating that chlorophyll content increases under SNP treatment, such as in wheat seedlings [60] and barley seedlings [61]. The measurement of chlorophyll fluorescence characteristics, which is practical, precise, and sensitive, is frequently employed to reflect the photosystem alterations that occur in plants under environmental stress [62]. Generally, plants grown under stress have a lower Fv/Fm than nonstressed plants [63], which is consistent with the performance of *S. polyrrhiza* (Fig. 1D). Compared with the control group, SNP treatment caused an increase in PSII activity in *S. polyrrhiza*, which is indicated by the lower Fv/Fm and higher ABS/RC and DIo/RC. The increase in ABS/RC in *S. polyrrhiza* under SNP treatment can be explained by a decrease in the number of active reactive centres (RCs) of PSII, which might serve as a defence mechanism to reduce the burden to its systems under stress. These findings are consistent with previous reports on *T. grandis* [57]. Antenna proteins are essential for absorbing solar energy and for photoprotection under stress [64]. These proteins assume a conformation that allows them to release extra energy from excitation as heat when plants are exposed to stress that could cause photooxidative damage [65]. We found that SNP treatment downregulated the expression levels of *LHCA4*, *LHCA5*, *LHCA6*, *LHCB2.1*, and *LHCB5*. Similar results were also reported by a previous study in alfalfa seedlings [21]. Therefore, we propose that SNP reduced photosynthesis by downregulating the expression levels of *LHCA4*, *LHCA5*, *LHCA6*, *LHCB2.1*, and *LHCB5*.

Starch is one of the major storage compounds of duckweed and usually accumulates under stress conditions, which is an adaptive strategy gained during the long evolutionary process to enable the plant to survive adverse environments [30]. The starch content reached 4.57 mg/g of fresh weight in the 0.025 mM SNP-treated samples and 1.83 mg/g of fresh weight in the control samples (Fig. 2C). As a result, the total starch accumulation in the treated samples was 1.5 times higher than that in the control samples. Starch accumulation is closely related to starch synthesis and metabolism. Two tactics are generally used to increase starch accumulation: increasing the amount of substrate from other metabolic pathways used for starch and sucrose metabolism or decreasing the pace at which starch is degraded [30]. AGPase is essential for controlling the accumulation of starch in plants [66]. In our study, the RNA-seq results showed that the expression of half of the key AGPase enzyme-encoding genes in the starch biosynthesis pathway was upregulated under SNP stress, while that of the other half was downregulated. Previous studies have shown that under conditions of nutritional starvation, the starch content and the expression of the AGPase gene in *Landoltia punctata* significantly increase [67]. We hypothesize that starch accumulation is also related to the decreased starch degradation in *S. polyrrhiza*. β-amylase is considered the main hydrolytic enzyme for breaking down starch granules. Previous studies have shown that the β-amylase gene is downregulated in *Landoltia punctata*, *L. turionifera*, kiwifruit and alfalfa [21, 68–70], which is consistent with our results, suggesting that it probably plays an important regulatory role in starch accumulation.

Overproduction of ROS during abiotic stress is typically observed, and this might result in some damage that eventually leads to oxidative stress [59]. In this study, we further explored the effects of SNP on the oxidative damage of *S. polyrrhiza*. As a result of the SNP treatments, the MDA levels in *S. polyrrhiza* increased, and ROS accumulation was observed, indicating oxidative damage. This is because elevated ROS levels may result in oxidative stress, which causes membrane damage and lipid peroxidation, and MDA can been used as a marker of this damage [71]. ROS-scavenging enzymes are crucial components of the plant defence mechanism in response to ROS production. Antioxidant enzymes and antioxidants synthesized in plants can remove excess ROS in response to adversity. In regard to enzymatic antioxidants, the enzyme SOD serves as the first line of defence by converting O^2-^ into H_2_O_2_, which is then metabolized by the enzymes APX, POD, and CAT [72]. SOD activity significantly increased at SNP treatment concentrations ranging from 0 mM to 0.01 mM compared to the control group. From 0.025 mM to 1.0 mM SNP, the SOD activity showed a downwards trend. The RNA-seq results showed that the gene expression of *S. polyrrhiza* SOD was lower after 0.025 mM SNP treatment. Although SOD activity was unchanged after 3 days of treatment with 0.025 mM SNP, we speculate that SOD activity might also show a downwards trend after treatment with 0.025 mM SNP for more than 3 days. We speculate that the change in SOD gene expression precedes SOD activity. The POD activity significantly increased at SNP treatment concentrations ranging from 0.025 mM to 0.5 mM compared to the control group. The RNA-seq results showed that nine of the seventeen POD-encoding transcripts were upregulated, while eight genes were downregulated under 0.025 mM SNP treatment. Therefore, the increased in POD activity is very understandable. This effect may be attributed to differences in gene expression regulation. In addition, the enzymatic properties of POD isoenzymes may be differ. A study of *S. polyrrhiza* indicated that enhancing antioxidative enzyme activity, such as that of POD and CAT, confers resistance to salt stress [73, 74]. In addition, the expression of genes related to antioxidants, such as *GPX*, *GST*, *MDAR*, *APX*, *LOX*, and *AOX*, was upregulated by SNP treatment. Research has shown that the expression of genes such as *GPX* was upregulated in SNP-treated *Ganoderma lucidum* mycelia [47].

The content of flavonoids such as orientin, vitexin, luteolin-7-O-glucoside and proanthocyanidins showed a significant downwards trend after rising first under SNP treatment. Transcriptome analysis showed that after SNP treatment, DEGs were highly enriched in phenylpropanoid metabolism (Fig. 10). Naringenin is one of the crucial substrates for the synthesis of flavonoids. Moreover, DEGs that encode the key enzymes in naringenin synthesis, such as *PAL*, *C4H*, *4CL*, *CHS*, and *CHI* had high expression levels under 0.025 mM SNP. Therefore, we speculate that these genes were upregulated, which may have led to an increase in naringenin biosynthesis. Naringenin can be catalysed by F3′H to produce flavonoids such as orientin, vitexin, and luteolin-7-O-glucoside. Meanwhile, the expression of *F3′H* was higher than 70 FPKM. This possibly explains why *S. polyrrhiza* under 0.025 mM SNP treatment accumulated flavonoids. In addition, naringenin can be catalysed by F3H, DFR and ANS to produce proanthocyanidins. Simultaneously, the expression of genes such as *F3H*, *DFR*, and *ANS* was also upregulated. This may be the main reason why *S. polyrrhiza* treated could accumulate proanthocyanidins under 0.025 mM SNP treatment. Overall, we speculate that flavonoids accumulated through the high expression of flavonoid-related genes, which was consistent with the observation in *Landoltia punctata* under nutrient starvation [75]. Furthermore, in the 0.01 and 0.025 mM SNP treatments, purple colouration accumulated on the underside of the frond, whereas the control group showed no discernible changes. It has been reported that the reddish-purple tint on the underside of its fronds is a result of anthocyanin production [75]. In our study, the genes encoding anthocyanin synthesis, such as *F3H*, *DFR*, *ANS*, and *UFGT* were upregulated. We speculate that SNP treatment may have contributed to anthocyanin accumulation in *S. polyrrhiza*, which was consistent with the observation in *Landoltia punctata* under nutrient starvation [75].

*S. polyrrhiza* is a promising chassis plant. It is important to monitor the metabolic flux changes in *S. polyrrhiza*. Previous studies have indicated that this plant responds to various abiotic stresses, such as nitrogen starvation [30], maleic hydrazide [41], and uniconazole [76], and the metabolic flux is mainly directed into the starch branch. Therefore, starch biosynthesis was enhanced. This may be explained as a stress escape or stress avoidance response to complete the life cycle in advance by storing most carbon nutrients and energy in starch [75]. Our study found that after treatment of *S. polyrrhiza* with 0.025 mM SNP, the metabolic flux was partly directed to the starch branch and partly to the flavonoid branch. The production of flavonoids, which are essential for plant defence against pathogens [77], can partially explain why *S. polyrrhiza* is rarely infected by pathogens.

Our study provides insights for further development of *S. polyrrhiza* as a chassis plant for the synthesis of secondary metabolites.

## Conclusions

SNP (0.025 mM) can effectively induce starch and flavonoid accumulation in *S. polyrrhiza*. Concretely, SNP may induce starch accumulation in *S. polyrrhiza* by downregulating the expression of key genes involved in starch degradation, while flavonoid accumulation in *S. polyrrhiza* may be induced by upregulating the expression of key genes involved in flavonoid biosynthesis. Moreover, SNP could promote the expression of some genes participating in starch and flavonoid accumulation, probably through the regulation of some TFs. These results suggest that after treatment of *S. polyrrhiza* with 0.025 mM SNP, the metabolic flux may be partly directed to the starch biosynthesis branches and partly into the flavonoid biosynthesis branches.

This provides a method to increase *S. polyrrhiza* starch and flavonoid accumulation in a selective manner. The genes regulated by SNP treatment would provide good candidates for improving the flavonoid content by genetic engineering in *S. polyrrhiza*.

## Methods

### Plant materials and treatments

*Spirodela polyrrhiza* strain P143 plants were described in the previous paper [78] and preserved at the Prof. Yong Wang’ s Lab. Plants of *S. polyrrhiza* were grown aseptically on DATKO medium as described by Wang and Kandeler [78] under a 16 h light/8 h dark cycle with a light intensity of ~ 45 µmolm^−2^s^−1^ at the plant level. The temperature of the culture room was maintained at 22 ± 2 °C. *S. polyrrhiza* plants were transferred to fresh medium every 10 days to minimize the effect of nutrient shortage. Plants were precultured under identical growth conditions for 8 days and then divided into eight groups as follows: (1) control (CK), without any treatment; (2) 0.01 mM SNP treatment; (3) 0.025 mM SNP treatment; (4) 0.05 mM SNP treatment; (5) 0.1 mM SNP treatment; (6) 0.3 mM SNP treatment; (7) 0.5 mM SNP treatment; and (8) 1.0 mM SNP treatment. Three biological replicates were performed. SNP was dissolved in distilled water, filter-sterilized and then added to DATKO medium. Plant samples were collected 1, 2, and 3 days after SNP treatment for measurement of NO content. Only the samples collected on the 3^rd^ day were used for the analysis of other physiological parameters. The starch content showed a significant upwards trend within the range of 0-0.025 mM SNP. However, starch content showed a significant downward trend within the range of 0.025-1.0 mM SNP. Therefore, we selected five representative concentrations, namely, 0, 0.01, 0.025, 0.1, and 0.5 mM SNP, for determination of the flavonoid content.

### Photosynthetic pigments contents and chlorophyll fluorescence

The photosynthetic pigment content was determined according to the method described by Arnon with some modifications [79]. Approximately 0.05 g (fresh weight) of sample was macerated in 3 mL of absolute ethanol, placed in the dark for 24 h at 28 °C, and centrifuged at 5000 × g for 5 min. The supernatant was collected, and the absorbance of chlorophyll a, b and carotenoids was recorded at 663, 645, and 470 nm, respectively. The PSII parameters were analysed using a pulse-amplitude modulation chlorophyll fluorometer (Handy-PEA, Hansatech, England). Measurements were performed with well-developed leaves treated with different concentrations of SNP, with three measurements per replicate. Each leaf was measured after dark adaptation for 30 min.

### Assays of physiological parameters

Fresh and dry weights were measured with a digital balance. The activities of SOD, CAT and POD were determined using enzyme activity kits (Beijing SolarBio Science & Technology Co., Ltd., Beijing, China). The contents of starch, soluble protein, MDA and H_2_O_2_ were determined using a content measurement kit (Beijing SolarBio Science & Technology Co., Ltd., Beijing, China); the proanthocyanidin content was determined by the Luthar and Kreft method [80]. Approximately 0.05 g of sample was macerated in 5 mL 60% ethanol, ultrasonicated at 30 °C for 30 min, and centrifuged at 5000 × g for 10 min at 25 °C. An aliquot of 1 mL of supernatant was mixed with 2 mL of 4% vanillin solution and 1 mL of concentrated hydrochloric acid for a 20 min reaction at room temperature, and the absorbance was measured at 530 nm.

### Determination of orientin, vitexin and luteolin-7-O-glucoside content

The dry materials were ground to a fine powder. Approximately 50 mg of powder was extracted in 5 mL of methanol by ultrasonication at 50 °C for 50 min, followed by centrifugation. Samples were separated using ultrahigh-performance liquid chromatography (UHPLC) with an ACQUITYUPLCBEH-C18 (2.1 mm × 50 mm, 1.7 μm, Waters). In the solvent system, mobile phase A was 0.1% (v: v) formic acid, and mobile phase B was methyl-alcohol. The gradient conditions applied were as follows: 0~7 min, 25% ~ 40% B; 7~10 min, 40% ~ 50% B; 10~11 min, 50% ~ 40% B; 11~12 min, 40% ~ 25% B; 12~14 min, 25% B. The flow rate was set at 0.3 mL/min, and the injection volume was 1 μL.

### RNA-seq and bioinformatics analysis

As described above under Plant materials and treatments, there were 3 flasks of repeat cultures for the control and 0.025 mM SNP treatment samples prepared for RNA-Seq. Three individual biological replicates (6 samples in total) were used for transcriptome analysis of *S. polyrrhiza*. Total RNA samples were extracted. The libraries were prepared using mRNA isolated from total RNA using Dynabeads oligo (dT). Then, the enriched mRNA fragments were randomly cleaved into short fragments of 200 nt-700 nt by adding fragmentation buffer, and the obtained short fragmented mRNA was used as the template. First-strand cDNA was generated using random hexamer primers. Second-strand cDNA was generated using buffer, dNTPs, RNaseH, and DNA polymerase I (Invitrogen). The cDNA fragments were then purified with a QiaQuick PCR extraction kit, the ends were repaired, poly(A) was added, and the fragments were ligated to Illumina sequencing adapters. Finally, the ligation products were size-selected using agarose gel electrophoresis, PCR amplified, and subjected to RNA-seq using an Illumina platform from Gene Denovo Biotechnology (Guangzhou, China).

Raw reads obtained from Illumina sequencing were further filtered to obtain high-quality clean reads by removing reads containing adapters, reads with an N ratio greater than 10%, reads containing a single base, and low-quality reads. De novo assembly of clean reads was performed using Trinity [81]. The fragments per kilobase of transcript per million mapped reads (FPKM) method was used to determine the expression levels of each gene. In this study, the level of differential expression for each transcript, with a criterion of |log2(fold-change)| >1 and FDR < 0.05 at different growth stages, was employed to identify DEGs. DEGs were subjected to enrichment analysis against the GO and KEGG databases to identify changes in biological functions [82–84].

### Quantification of gene expression levels

Total RNA was isolated from the CK and 0.025 mM SNP*-*treated *S. polyrrhiza* samples using an Eastep Super Total RNA Extraction Kit (Promega, Shanghai) according to the manufacturer’s protocol. The integrity of the total RNA was examined using 1.0% agarose gel electrophoresis. Primer sequences for selected DEGs (Additional file 11: Table S8) were designed using Primer Premier 5 and synthesized by Sales Genomics CN (Tianjin, China). First-strand cDNA was synthesized using a FastKing RT Kit containing gDNase (TIANGEN Biotech, China) with one cycle at 42 °C for 15 min and then 95 °C for 3 min. qRT‒PCR for gene expression analysis was carried out using SuperRealPreMix Plus (SYBR Green) (TIANGEN Biotech, China) with one cycle at 95 °C for 15 min followed by 40 cycles of 95 °C for 10 s and 60 °C for 30 s. Actin was used as a reference control gene. The 2^−ΔΔCt^ method [85] was used to calculate relative gene expression.

### Statistical analysis

All measurements were performed using three biological replicates. Statistical analysis was performed with GraphPad Prism 8.0 software, and significance analysis was conducted with one-way ANOVA with multiple comparisons (*P* < 0.05 or *P* < 0.01).

### Supplementary Information

**Additional file 1:** **Figure S1.** Phenotypic changes of *S. polyrrhiza* after SNP treatment.

**Additional file 2:** **Figure S2. **Species distribution of Nr annotation.

**Additional file 3:** **Figure S3. **The relative expression levels of seventeen selected DEGs were compared by RNA-seq and qRT-PCR.

**Additional file 4**: **Table S1. **The DEGs were identified as transcription factor.

**Additional file 5:** **Table S2. **The DEGs were identified as photosynthesis.

**Additional file 6:** **Table S3. **The DEGs were identified as starch metabolism.

**Additional file 7:** **Table S4. **The DEGs were identified as antioxidant.

**Additional file 8:** **Table S5. **The DEGs were identified as flavonoids biosynthesis pathways.

**Additional file 9:** **Table S6. **DEGs involved in quantitative real-time PCR validation.

**Additional file 10:** **Table S7.** DEGs involved in NO biosynthetic and signal transduction.

**Additional file 11:** **Table S8. **Primers used for QRT-PCR analysis.

## Data Availability

The transcriptome datasets supporting the conclusions of this study have been uploaded to the National Center for Biotechnology Information (accession number: PRJNA922734) and can be accessed using the following link https://www.ncbi.nlm.nih.gov/bioproject/?term=PRJNA922734. The other supporting data are included as supplemental files.

## Abbreviations

SNP: Sodium nitroprusside
NO: Nitric oxide
Fv/Fm: PSII maximum photochemical conversion efficiency
ABS/RC: Absorption flux per reaction center
DIo/RC: Dissipation energy per reaction center
MDA: Malondialdehyde
H_2_O_2_: Hydrogen peroxide
SOD: Superoxide dismutase
POD: Peroxidase
CAT: Catalase
NCBI: National Center for Biotechnology Information
KEGG: Kyoto Encyclopedia of Genes and Genomes
DEGs: Differentially expressed genes
TF: Transcription factor
FPKM: Fragments per kilobase of transcript per million mapped reads
ROS: Reactive oxygen species
