# Supplementary figures and images for: Transcriptomic and physiological analysis of the response of Spirodela polyrrhiza to sodium nitroprusside
Source: BMC Plant Biol. 2024 Feb 8;24:95. doi: 10.1186/s12870-024-04766-6 (PMC10851477; doi:10.1186/s12870-024-04766-6)

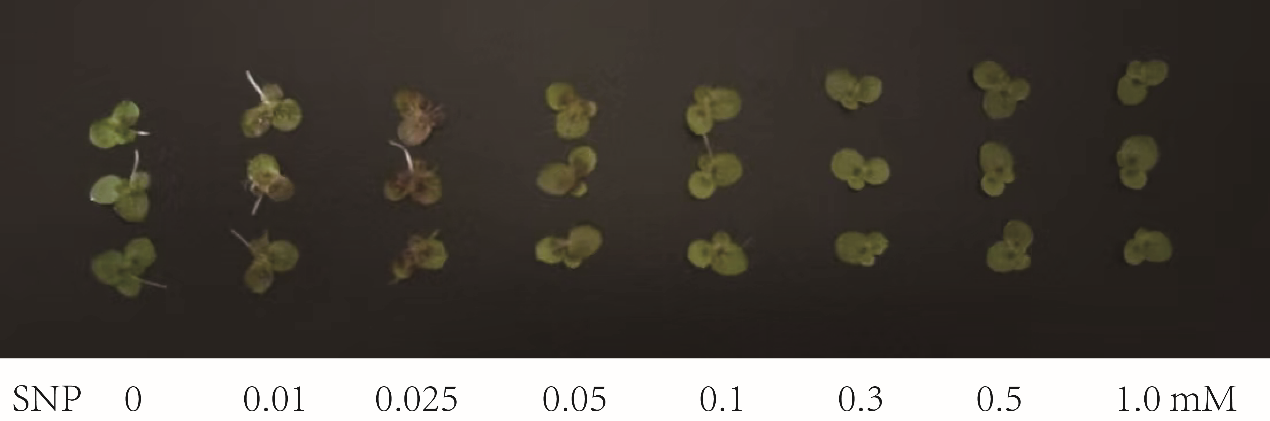


**Figure S1.** Phenotypic changes of *S. polyrrhiza* after SNP treatment.

Supplement: Supplementary file 1 — Additional file 1: Figure S1. Phenotypic changes of S. polyrrhiza after SNP treatment. [file 12870_2024_4766_MOESM1_ESM.docx]

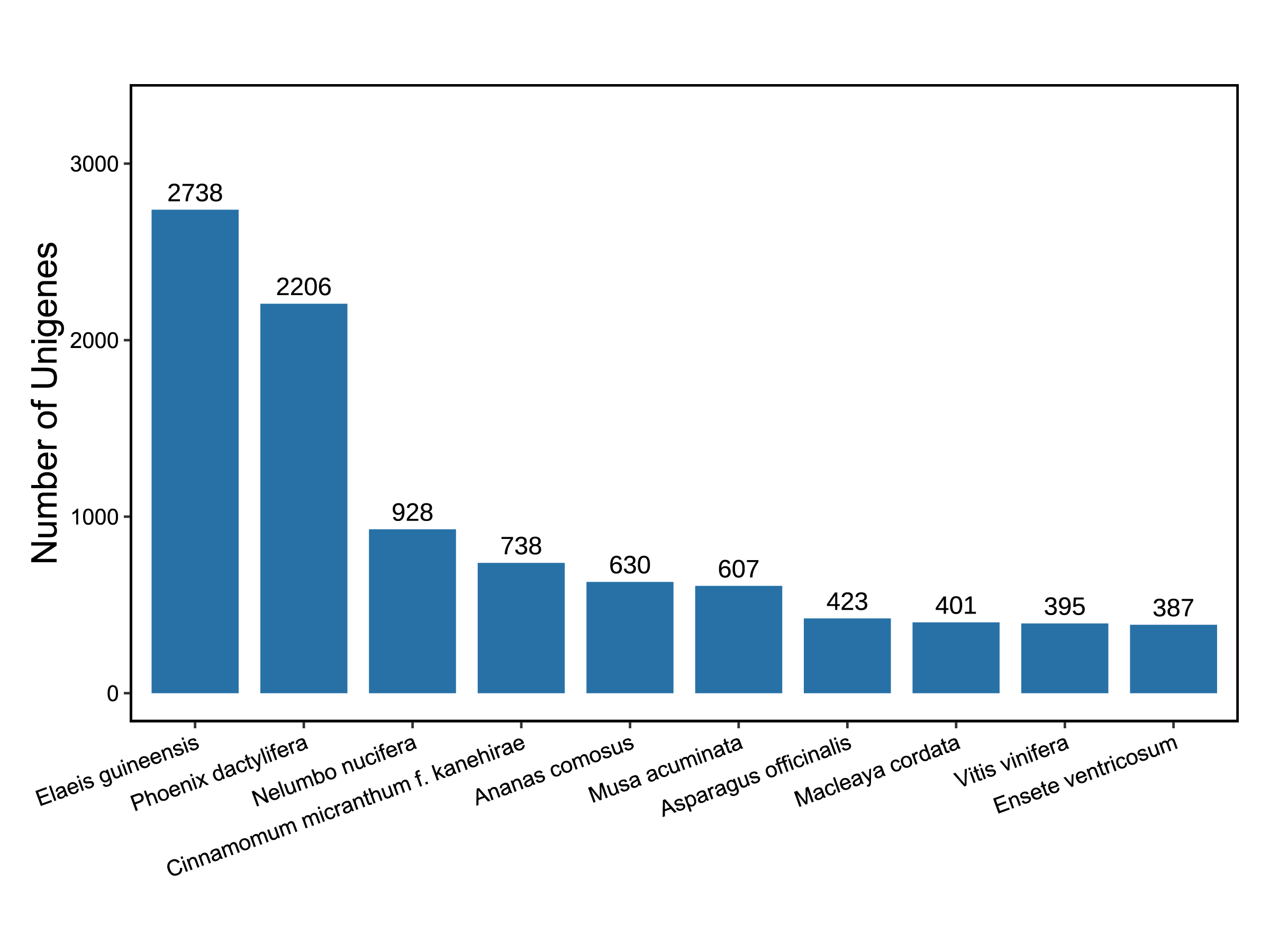


**Figure S2.** Species distribution of Nr annotation

Supplement: Supplementary file 2 — Additional file 2: Figure S2. Species distribution of Nr annotation. [file 12870_2024_4766_MOESM2_ESM.docx]
