# Supplementary material for: Transcriptomic and physiological analysis of the response of Spirodela polyrrhiza to sodium nitroprusside
Source: BMC Plant Biol. 2024 Feb 8;24:95. doi: 10.1186/s12870-024-04766-6 (PMC10851477; doi:10.1186/s12870-024-04766-6)
Supplement: Supplementary file 3 — Additional file 3: Figure S3. The relative expression levels of seventeen selected DEGs were compared by RNA-seq and qRT-PCR. [file 12870_2024_4766_MOESM3_ESM.docx]

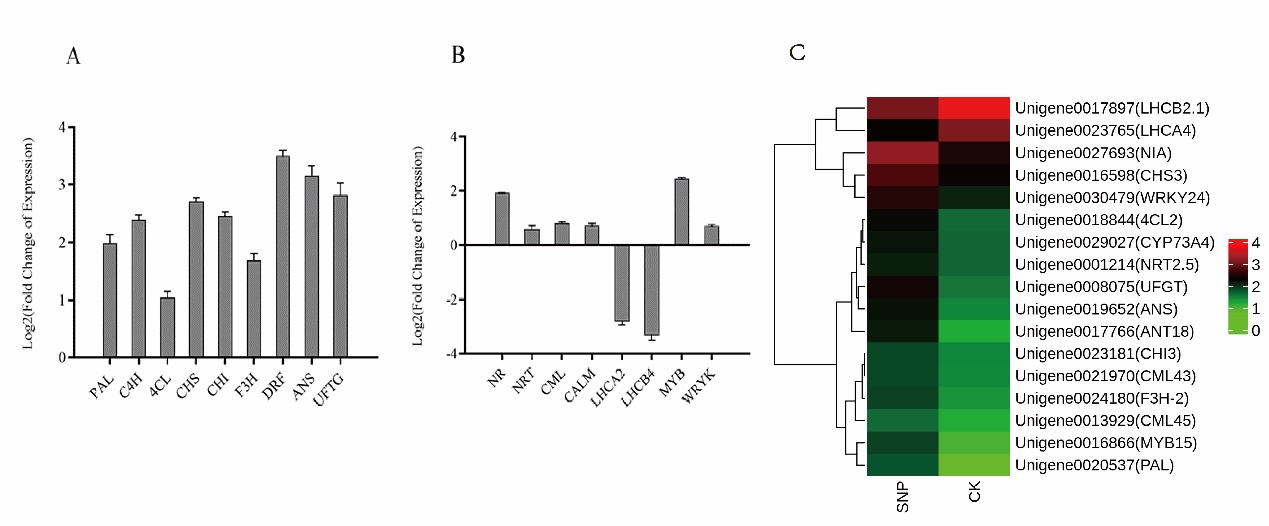


**Figure S3.** The relative expression levels of seventeen selected DEGs were compared by RNA-seq and qRT-PCR. A flavonoid biosynthesis pathway gene expression level; B other pathway gene expression levels; C expression pattern of genes involved in qRT-PCR validation. Data are presented as means ± standard deviations (n = 3)
